# First Draft genome sequence of strain MF0224, representing an unclassified *Fusobacterium*-related lineage isolated from coastal sediments of Puducherry, India

**DOI:** 10.1016/j.dib.2026.113126

**Published:** 2026-08-03

**Authors:** Munisamy Prathaban, Ragothaman Prathiviraj, Murugesan Sobanaa, S Hari Krishna Kumar, Rathinam Thiruppathi Venkadajalapathy Vimala, George Seghal Kiran, Joseph Selvin, Laurent Dufossé

**Affiliations:** a Carbon Capture and Utilization Laboratory, Department of Microbiology, Pondicherry University, Pondicherry 605014, India; b Division of Bioinformatics and Systems Biology, Department of Biosciences, Rajagiri College of Social Sciences (Autonomous), Rajagiri P.O., Kalamassery, Kochi 683104, India; c Microbial Genomics Laboratory, Department of Microbiology, Pondicherry University, Pondicherry 605014, India; d Department of Food Science and Nutrition, Pondicherry University, Pondicherry 605014, India; e Chemistry and Biotechnology of Natural Products, CHEMBIOPRO, Université de La Réunion, ESIROI Agroalimentaire, 15 Avenue René Cassin, CS 92003, CEDEX 9, F-97744 Saint-Denis, France

**Keywords:** Acetate metabolism, Carbon cycle, Comparative genomics, Coastal sediments, Draft genome sequence, *Fusobacterium*

## Abstract

This article presents the draft genome sequence of strain MF0224, an anaerobic bacterium representing an unclassified *Fusobacterium*-related lineage isolated from a coastal sediment sample collected in Puducherry, India. Whole-genome sequencing was performed using the Illumina paired-end sequencing platform and assembled into a draft genome of 2.02 Mb comprising 26 contigs with a genomic DNA G+C content of 28.19%. Genome annotation predicted 1889 coding sequences, 47 tRNA genes, and multiple genes associated with anaerobic carbon metabolism, acetate production, sulfur metabolism, volatile fatty acid metabolism, and carbohydrate-active enzymes. Genome quality assessment indicated high completeness with low contamination. Comparative genomic analysis using TYGS identified the strain as a potential novel species within the genus *Fusobacterium*, supported by low digital DNA–DNA hybridization (dDDH) values (∼20%) relative to currently described species. Average amino acid identity (AAI; 68.9%) supported its placement within the genus *Fusobacterium* while indicating substantial genomic divergence. Comparative genomic analysis also determined a percentage of conserved proteins (POCP) value of 65.9%, providing additional genomic characterization of strain MF0224. Phylogenetic analyses based on 16S rRNA gene sequences and whole-genome phylogeny consistently positioned strain MF0224 within the *Fusobacterium*-associated clade. The genome provides a valuable resource for investigating anaerobic carbon metabolism, acetate biosynthesis, sulfur-associated pathways, and the ecological adaptation of *Fusobacterium*-related bacteria in coastal sediment ecosystems.

Specification Table

**Table:** 

Subject	Applied Microbiology and Biotechnology
Specific subject area	Microbial genomics and comparative genomics
Type of data	Draft genome sequence, comparative genomic analysis, phylogenetic analysis
Data collection	Sediment samples were collected from coastal wetlands of Puducherry, India. Genomic DNA was extracted and sequenced using Illumina paired-end sequencing. Quality filtering was performed using FastQC and Fastp. Genome assembly was carried out using MEGAHIT, followed by annotation using Prokka and eggNOG mapper.
Data source location	*Fusobacterium* sp. MF0224 was isolated from sediment samples taken from the intertidal region of the Pondicherry coast in India (11.900,476° N, 79.812,477° E).
Data accessibility	Whole Genome data was deposited in the National Center for Biotechnology Information (NCBI) GenBank database with the following accession number NZ_JBJDSC000000000.1. The deposited draft genome data available at https://www.ncbi.nlm.nih.gov/nuccore/NZ_JBJDSC000000000.1
Related research article	None

## 1 Value of the Data

• The draft genome of strain MF0224 expands the genomic representation of environmentally associated *Fusobacterium*-related bacteria isolated from tropical coastal wetland ecosystems of India.
• The genome provides insight into acetate metabolism, volatile fatty acid biosynthesis, sulfur-associated pathways, and anaerobic fermentative metabolism relevant to carbon cycling in coastal sediments.
• Comparative genomic analyses revealed substantial divergence from currently described *Fusobacterium* species while maintaining conserved genus-level characteristics.
• The availability of genome assembly statistics, TYGS-based phylogenomics, ANI, AAI, and POCP analyses provides a useful resource for future taxonomic and ecological investigations involving anaerobic sediment-associated bacteria.
• Functional annotation may support future studies on anaerobic biotechnology, carbon conversion processes, and microbial adaptation in wetland environments.

## 2 Background

Members of the genus *Fusobacterium* are anaerobic Gram-negative bacteria widely distributed in animal-associated habitats, sediments, and oxygen-limited environments [1]. Several species within the genus possess versatile fermentative metabolisms capable of utilizing amino acids and organic substrates for the production of acetate and other volatile fatty acids [2,3]. These metabolic characteristics contribute significantly to anaerobic carbon turnover and nutrient cycling in natural ecosystems [4,5].

Coastal wetland sediments represent dynamic anaerobic environments characterized by fluctuating salinity, high organic matter content, sulfur-rich conditions, and intense microbial interactions [6,7]. Microorganisms inhabiting these environments often possess specialized metabolic pathways associated with fermentation, sulfur metabolism, stress adaptation, and anaerobic respiration [6,8]. Genome-resolved characterization of such microorganisms provide important insights into ecological adaptation and metabolic specialization within coastal sediment ecosystems [8,9].

In this study, strain MF0224 was isolated from coastal wetland sediments collected in Puducherry, India. Whole genome sequencing and comparative genomic analyses were performed to investigate its genomic features, phylogenetic placement, and metabolic potential. Genome-based taxonomy revealed that strain MF0224 represents a highly divergent lineage affiliated with the genus *Fusobacterium*, potentially representing a novel species-level taxon.

## 3 Data Description

**Fig. 1:**
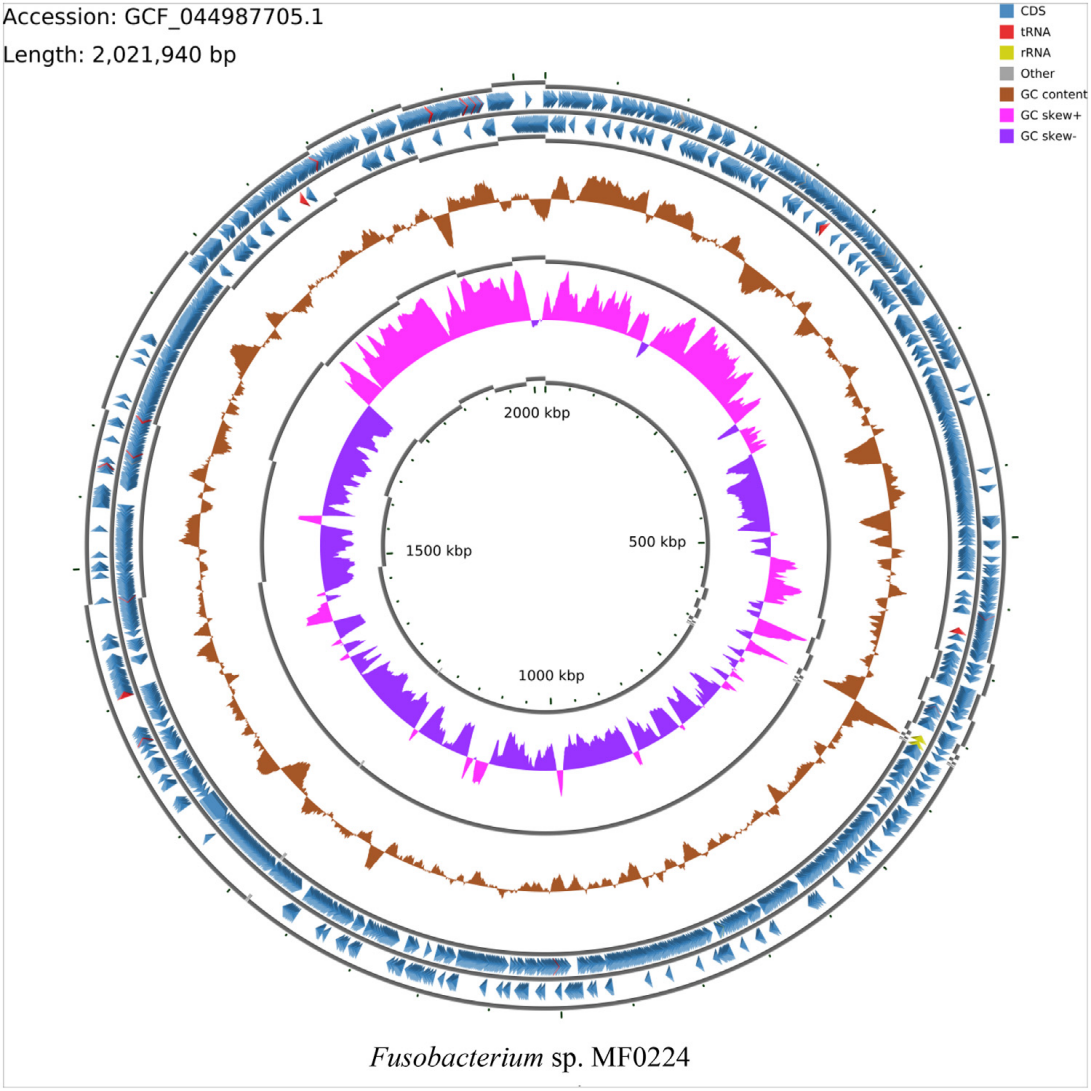
Circular genome map of *Fusobacterium* sp. MF0224. The circular representation illustrates the genomic architecture of strain MF0224 (GenBank accession: GCF_044987705.1), comprising a 2021,940 bp draft genome with a GC content of 28.19%. From the outermost to the innermost rings: coding sequences (CDSs) on the forward strand (blue), coding sequences on the reverse strand (blue), tRNA genes (red), rRNA genes (yellow), and other genomic features (gray). The brown ring represents GC content variation across the genome, while the innermost rings depict GC skew, with positive values shown in magenta and negative values shown in purple. The genome map highlights the distribution of coding regions and nucleotide composition, providing an overview of the genomic organization of strain MF0224.

This dataset reports the draft genome sequence of *Fusobacterium* sp. MF0224, an obligate anaerobic isolate of ecological importance due to its carbon cycle capabilities in coastal wetlands. The draft genome of strain MF0224 consists of 2021,940 bp assembled into 26 contigs with an average genomic DNA G+C content of 28.19%. Genome assembly statistics revealed an N50 value of 569,293 bp and an L50 value of 2, indicating good assembly continuity. Genome quality assessment using CheckM v1.2.3 indicated that the draft genome of *Fusobacterium* sp. MF0224 was of high quality, with an estimated completeness of 97.37% and a contamination level of 4.37%. The assessment was performed using the PGAP-predicted gene set and the *Fusobacterium*-specific marker lineage, supporting the reliability of the assembled genome for downstream comparative genomic and taxonomic analyses. Genome annotation predicted 1889 coding sequences (CDSs) and 47 tRNA genes (Table 1). To provide an overview of the genomic architecture, a circular genome map was generated (Fig. 1). The genome map illustrates the distribution of CDSs on both forward and reverse strands, along with tRNA, rRNA, and other genomic features. Coding sequences were distributed throughout the genome with no apparent large-scale genomic rearrangements, indicating a relatively conserved genomic organization. Analysis of genomic DNA G+C content revealed localized variations across the genome, reflecting regions of differential nucleotide composition, while the genomic DNA G+C skew profile exhibited alternating positive and negative regions that may correspond to replication-associated strand asymmetry. Collectively, the circular genome map provides a comprehensive visualization of gene distribution, nucleotide composition, and structural organization of *Fusobacterium* sp. MF0224, serving as a foundation for subsequent comparative genomic and functional analyses.

**Table 1:** Genome statistics of *Fusobacterium* sp. MF0224.

Features	Count
Contigs	26
G+C content	28.19%
Genome length (bp)	20,21,940
Contig L50	2
Contig N50	5,69,293
CDS	1889
tRNA	47
rRNA	3
Hypothetical proteins	663

Genome-based taxonomic analysis was performed using the Type (Strain) Genome Server (TYGS). TYGS identified strain MF0224 as a “potential new genospecies.” Pairwise digital DNA–DNA hybridization (dDDH) analysis showed low relatedness with all currently described type strains. The highest dDDH value was observed with *Fusobacterium perfoetens* ATCC 29250^T^ (dDDH_d4 = 20.2%), which is substantially below the accepted species delineation threshold of 70%.

Average amino acid identity (AAI) analysis demonstrated the highest similarity between strain MF0224 and *Fusobacterium perfoetens* ATCC 29250^T^ (AAI = 68.9%), while other *Fusobacterium* species exhibited AAI values ranging from approximately 59–62%. Percentage of conserved proteins (POCP) analysis revealed a value of 65.9% relative to closely related *Fusobacterium* species, supporting genus-level affiliation while indicating substantial genomic divergence.

**Fig. 2:**
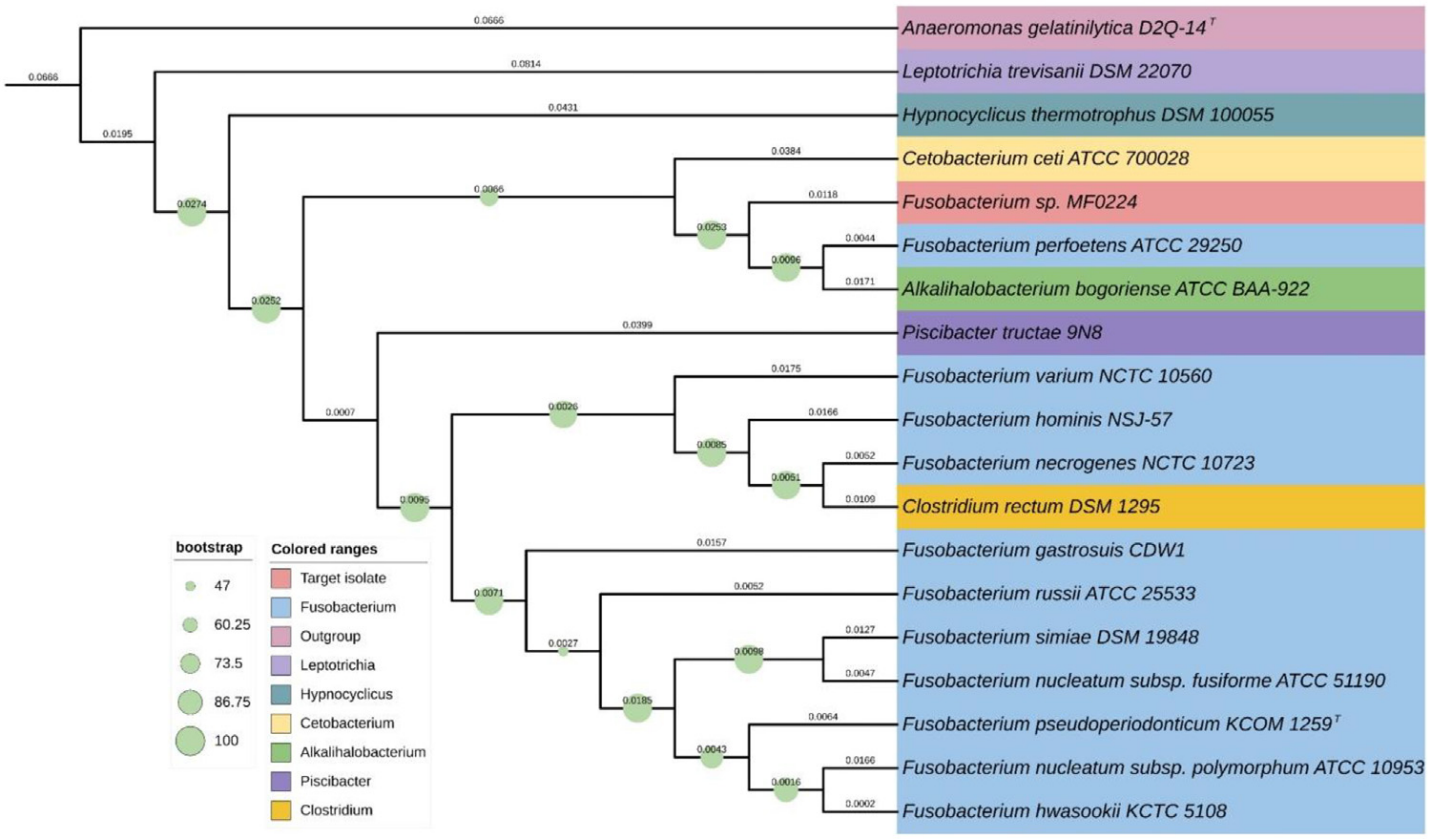
Phylogenetic tree based on 16S rRNA gene sequences showing the taxonomic position of strain MF0224 among representative members of the family Fusobacteriaceae and related taxa. Strain MF0224 is highlighted in pink and clusters within the *Fusobacterium*-associated lineage, showing closest phylogenetic affiliation with *Fusobacterium perfoetens* ATCC 29,250 and related species. Bootstrap support values are indicated by node symbols, and different colors represent distinct taxonomic groups included in the analysis.

**Fig. 3:**
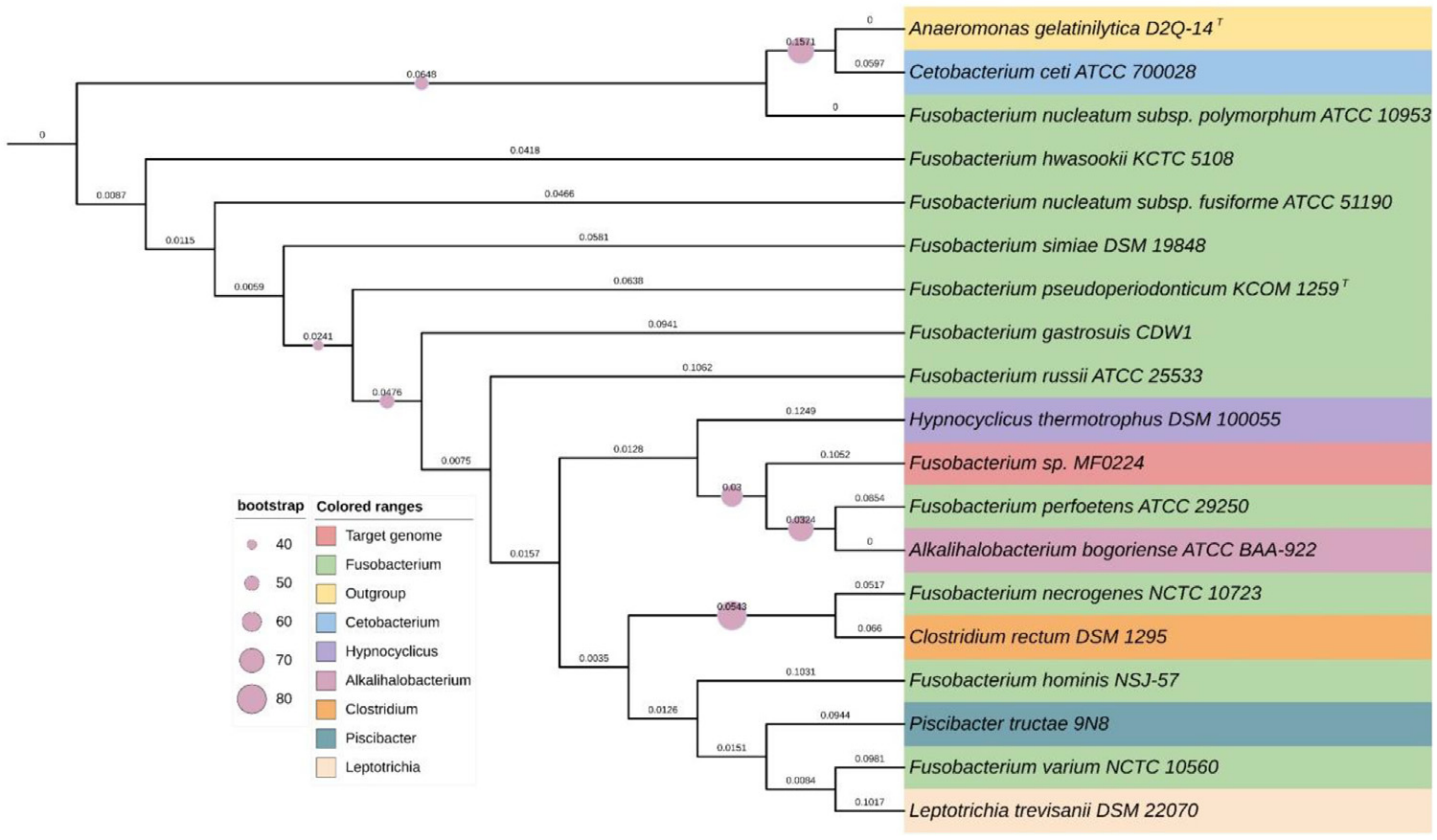
Whole-genome phylogenetic tree showing the evolutionary relationship of strain MF0224 with representative members of the family Fusobacteriaceae and related taxa. The phylogenomic tree was constructed using whole-genome sequence data. Strain MF0224 (highlighted in pink) clustered within the *Fusobacterium* lineage and exhibited the closest genomic relationship to *Fusobacterium perfoetens* ATCC 29,250. Bootstrap support values are represented by node symbols, and different colors indicate the major taxonomic groups included in the analysis.

Phylogenetic analyses based on both 16S rRNA gene sequences and whole-genome data were performed to determine the taxonomic position of strain MF0224 (Figs. 2 & 3). In both analyses, strain MF0224 clustered within the *Fusobacterium* lineage and exhibited the closest phylogenetic relationship to *Fusobacterium perfoetens* ATCC 29250^T^. The congruent clustering patterns obtained from 16S rRNA gene phylogeny and whole-genome phylogeny were consistent with genome-based taxonomic analyses, supporting the assignment of strain MF0224 to the genus *Fusobacterium*. Despite its affiliation with recognized *Fusobacterium* species, strain MF0224 occupied a distinct branch in both trees, indicating substantial evolutionary divergence from currently described taxa. These findings, together with the low dDDH values and divergent ANI, AAI, and POCP metrics, support the classification of strain MF0224 as a previously uncharacterized and potentially novel species-level lineage within the genus *Fusobacterium*.

Functional annotation identified multiple genes involved in acetate production and acetyl-CoA metabolism, indicating fermentative carbon metabolism consistent with anaerobic sediment-associated bacteria. The genome encodes a complete acetate production pathway, and acetyl-CoA metabolism (Table 2). The genome encoded a complete acetate-forming pathway comprising phosphate acetyltransferase (*pta; n* = 1) and acetate kinase (*ackA; n* = 1), which catalyze the conversion of acetyl-CoA to acetate with concomitant ATP generation. Genes involved in pyruvate oxidation, including pyruvate oxidoreductase (*porA/B/C/D; n* = 4) and pyruvate dehydrogenase complex subunits (*pdh; n* = 3), were also identified, indicating multiple routes for acetyl-CoA formation. Additional genes such as alanine dehydrogenase (*ald; n* = 2) suggest a link between amino acid catabolism and acetate biosynthesis. The genome further encoded several ferredoxin proteins (*n* = 5), oxidoreductases (*n* = 16), and alcohol/acetaldehyde dehydrogenases (*adhE*-like; *n* = 4), which are involved in electron transfer and redox balancing during anaerobic metabolism. A putative acetyl-CoA synthetase gene (*acs; n* = 1) and multiple membrane transporters (*n* = 21) associated with metabolite transport were also detected. Collectively, these findings indicate the genomic potential of strain MF0224 for acetate production and anaerobic fermentative metabolism, which may contribute to carbon cycling processes in coastal sediment environments. Genes associated with sulfur metabolism and stress adaptation suggest ecological specialization for coastal wetland environments characterized by fluctuating redox conditions and sulfur-rich sediments. The genome additionally encoded multiple hypothetical proteins that may contribute to environmental adaptation and anaerobic metabolism in coastal sediment ecosystems.

**Table 2:** Genes involved in acetate production and acetyl-CoA metabolism identified in the genome of *Fusobacterium* sp. MF0224. Gene copy numbers and predicted functions were determined based on Prokka annotation and eggNOG functional assignments. The identified genes represent key enzymatic steps associated with pyruvate conversion, acetyl-CoA formation, acetate biosynthesis, electron transfer, redox balance, and metabolite transport.

Gene Name	No. of Genes	Function
*pta* (phosphate acetyltransferase)	1	Converts acetyl-CoA → acetyl phosphate
*ackA* (acetate kinase)	1	Converts acetyl phosphate → acetate + ATP
*porA/B/C/D* (pyruvate:ferredoxin oxidoreductase)	4	Converts pyruvate → acetyl-CoA (anaerobic)
*pdh* complex (pyruvate dehydrogenase subunits)	3	Alternative pyruvate → acetyl-CoA route
*ald* (alanine dehydrogenase)	2	Converts alanine → pyruvate
ferredoxin (Fe-S proteins)	5	Electron transfer in anaerobic metabolism
oxidoreductases (general dehydrogenases)	16	Maintain NADH/redox balance
alcohol/acetaldehyde dehydrogenase	4	Links acetyl-CoA and redox metabolism
*acs* (acetyl-CoA synthetase)	1	Reversible acetate ↔ acetyl-CoA
*MFS/ABC* (membrane transporters)	21	Export acetate and metabolites

Recent genome-based taxonomic studies have highlighted the importance of integrating whole-genome sequencing, phylogenomic inference, and overall genome-relatedness indices (ANI, AAI, and dDDH) for the characterization of novel bacterial taxa. Comparative genomic analyses of bacterial isolates from marine and mangrove environments demonstrated that genome-resolved approaches provide robust evidence for species delineation while simultaneously revealing ecological adaptations and metabolic potential associated with coastal and mangrove ecosystems. Similar to these studies, the genome of *Fusobacterium* sp. MF0224 expands the genomic diversity of sediment-associated bacteria by providing genome-scale evidence supporting its classification as a potentially novel species within the genus *Fusobacterium* and offers insights into metabolic functions related to anaerobic carbon metabolism and ecological adaptation in coastal wetland sediments [[10], [11], [12], [13]].

## 4 Experimental Design, Materials and Methods

### 4.1 Sample collection and isolation

Sediment samples were collected from coastal wetlands of Puducherry, India, under aseptic conditions [14]. Samples were transported to the laboratory under cold conditions and processed immediately for anaerobic enrichment and bacterial isolation. Anaerobic enrichment cultures were prepared using suitable anaerobic media with acetate as carbon source under nitrogen atmosphere. Pure cultures were obtained through serial dilution and roll tube method [14,15]. Strain MF0224 was used for genome sequencing based on its distinct anaerobic growth characteristics.

### 4.2 *Genomic DNA extraction from fusobacterium* sp. MF0224

*Fusobacterium* sp. MF0224 was cultivated under anaerobic conditions in an appropriate medium at 37 °C until the mid-logarithmic growth phase. Cells were harvested by centrifugation at 8000 × g for 10 min, and genomic DNA was extracted using a HiMedia DNA extraction kit (HiMedia, India) following the manufacturer's instructions [16].

### 4.3 Whole-Genome sequencing, assembly, and annotation

The genomic DNA of *Fusobacterium* sp. MF0224 was sequenced at Macrogen Inc. (Seoul, South Korea) using the Illumina HiSeq platform with paired-end 150 bp chemistry (PE150). Raw sequencing reads were subjected to quality assessment using FastQC v0.11.9 to evaluate sequence quality, genomic DNA G+C distribution, adapter contamination, and duplication levels [17]. Adapter sequences and low-quality reads were removed using Fastp to improve read quality for downstream analyses [18].

High-quality filtered reads were assembled *de novo* using the MEGAHIT assembler, a computationally efficient tool optimized for short-read genome assembly [19,20]. Assembly quality metrics, including genome size, genomic DNA G+C content, contig statistics, N50, and L50 values, were evaluated using Quality Assessment Tool for Genome Assemblies (QUAST) [21]. Genome completeness and contamination were further assessed using CheckM v1.2.3 with lineage-specific marker genes to estimate assembly quality and genome integrity [22].

The structural organization and genomic feature distribution of *Fusobacterium* sp. MF0224 were visualized using a circular genome map generated through the Proksee web server. Genome annotation and gene prediction were performed using Prokka v1.14.6 and eggNOG-mapper v2.1.13, enabling the identification of coding sequences (CDSs), ribosomal RNA genes, transfer RNA genes, and functional elements, together with preliminary functional assignments [23]. The annotated genome was subsequently used for comparative genomic, phylogenetic, and functional analyses.

### 4.4 Phylogenetic and genome-based taxonomic analyses

The assembled genome of *Fusobacterium* sp. MF0224 was submitted to the Type (Strain) Genome Server (TYGS; https://tygs.dsmz.de) for genome-based taxonomic assessment using the latest analytical framework [24]. Taxonomic nomenclature and reference information were obtained from the List of Prokaryotic Names with Standing in Nomenclature (LPSN; https://lpsn.dsmz.de) [23].

The closest phylogenetic relatives were identified using two TYGS-integrated approaches: (i) MASH-based comparison of the query genome against all available type strain genomes to identify the ten nearest neighbors based on minimum MASH distances [25], and (ii) extraction of 16S rRNA gene sequences using RNAmmer [26], followed by BLAST comparison against 23,787 type strain sequences. The top 50 matches were subjected to distance estimation using Genome BLAST Distance Phylogeny (GBDP) with the coverage algorithm and distance formula d5 [27]. The ten most closely related strains were selected for subsequent analyses.

Phylogenomic inference was performed using the TYGS platform based on GBDP distances calculated with the trimming algorithm and distance formula d5. To ensure robustness of the inferred phylogeny, 100 distance replicates were generated for each pairwise comparison [28]. Digital DNA–DNA hybridization (dDDH) values and their corresponding confidence intervals were calculated according to the Genome-to-Genome Distance Calculator (GGDC 4.0) recommendations [26,28]. In addition, average nucleotide identity (ANI), average amino acid identity (AAI), and percentage of conserved proteins (POCP) analyses were performed to further evaluate the genomic relatedness and taxonomic position of strain MF0224 relative to closely related members of the genus *Fusobacterium*.

## Limitations

The genome presented in this study is a draft assembly generated using short-read sequencing and therefore remains fragmented into multiple contigs. Functional annotations and metabolic predictions are based on bioinformatic analyses and require experimental validation. Although genome-based taxonomic analyses support the classification of strain MF0224 as a potentially novel species within the genus *Fusobacterium*, additional phenotypic characterization is necessary for formal taxonomic description.

## Ethics Statement

This study did not involve any experiments on human participants or animals. All data were obtained through computational analyses. Hence, ethical approval was not required for this work.

## CRediT Author Statement

**Munisamy Prathaban:** Validation, Conceptualization, Methodology**,** Writing – original draft, Writing – review & editing; **Ragothaman Prathiviraj:** Visualization, Writing – review & editing, Data curation; **Murugesan Sobanaa:** Writing – original draft, Methodology, Writing – review & editing; **S. Hari Krishna Kumar**: Visualization, Writing – review & editing, Data curation; **Rathinam Thiruppathi Venkadajalapathy Vimala**: Visualization, Writing – review & editing; **George Seghal Kiran**: Visualization, Writing – review & editing; **Joseph Selvin**^:^ Supervision, Administration, resource, Writing – review & editing **Laurent Dufossé:** Supervision, Conceptualization, Writing – original draft, Writing – review & editing.

## Declaration of Generative AI and AI-Assisted Technologies in the Writing Process

During the preparation of this work, the authors used Chat GPT and Quilbot for paraphrasing and language improvement.

## Data Availability

NCBIwww.ncbi.nlm.nih.gov/datasets/genome/GCF_044987705.1 (Original data)

## Appendix Supplementary materials

Image, application 1

Image, application 2

Image, application 3

Image, application 4
