# Supplementary material for: First Draft genome sequence of strain MF0224, representing an unclassified Fusobacterium-related lineage isolated from coastal sediments of Puducherry, India
Source: Data Brief. 2026 Aug 3;68:113126. doi: 10.1016/j.dib.2026.113126 (PMC13487623; doi:10.1016/j.dib.2026.113126)
Supplement: Supplementary file 1 [file mmc1.pdf]

TYPE (STRAIN) GENOME SERVER

JOB SUMMARY

PRINT DATE:	2026-04-23 13:14:59 +0200
JOB ID:	49222de3-65b0-45fc-8898-1a47b25a2fd3
RESULT PAGE:	https://tygs.dsmz.de/user_results/show?guid=49222de3-65b0-45fc-8898-1a47b25a2fd3

Table 1: Phylogenies

Publication-ready versions of both the genome-scale GBDP tree and the 16S rRNA gene sequence tree can be customized and exported either in SVG (vector graphic) or PNG format from within the phylogeny viewers in your TYGS result page. For publications the **SVG format is recommended** because it is lossless, always keeps its high resolution and can also be easily converted to other popular formats such as PDF or EPS. Please follow the link provided above!

Table 2: Identification

The below list contains the result of the TYGS species identification routine.

Explanation of remarks that might occur in the below table:

remark [R1]: The TYGS type strain database is automatically updated on an almost daily basis. However, if a particular type strain genome is not available in the TYGS database, this can have several reasons which are detailed in the FAQ. You can request an extended 16S rRNA gene analysis via the 16S tree viewer found in your result page to detect **not yet genome-sequenced** type strains relevant for your study.

remark [R2]: > 70% dDDH value (formula d_4) and (almost) minimal dDDH values for gene-content formulae d_0 and d_6 indicate a potentially unreliable identification result and should thus be checked via the 16S rRNA gene sequence similarity. Such strong deviations can, in principle, be caused by sequence contamination.

remark [R3]: G+C content difference of > 1 % indicates a potentially unreliable identification result because within species G+C content varies no more than 1 %, if computed from genome sequences (PMID: 24505073).

Strain	Conclusion	Identification result	Remark
**'Fusobacterium sp. MF0224'**	potential new species		see [R1]

Table 3: Pairwise comparisons of user genomes vs. type-strain genomes

The following table contains the pairwise dDDH values between your user genomes and the selected type-strain genomes. The dDDH values are provided along with their confidence intervals (C.I.) for the three different GBDP formulas:

- formula d_0 (a.k.a. GGDC formula 1): length of all HSPs divided by total genome length
- formula d_4 (a.k.a. GGDC formula 2): sum of all identities found in HSPs divided by overall HSP length
- formula d_6 (a.k.a. GGDC formula 3): sum of all identities found in HSPs divided by total genome length

Note: Formula d_4 is independent of genome length and is thus robust against the use of incomplete draft genomes. For other reasons for preferring formula d_4, see the FAQ.

Query	Subject	d_0	C.I. d_0	d_4	C.I. d_4	d_6	C.I. d_6	Diff. G+C Percent
**'Fusobacterium sp. MF0224.fa'**	*Alkalihalobacterium bogoriense* ATCC BAA-922	12.5	[9.9 - 15.8]	49.2	[46.6 - 51.8]	12.9	[10.6 - 15.7]	8.49
**'Fusobacterium sp. MF0224.fa'**	*Fusobacterium hominis* NSJ-57	13.5	[10.8 - 16.9]	21.0	[18.8 - 23.5]	13.8	[11.4 - 16.6]	0.86
**'Fusobacterium sp. MF0224.fa'**	*Leptotrichia trevisanii* DSM 22070	12.6	[9.9 - 15.8]	20.4	[18.2 - 22.9]	13.0	[10.7 - 15.7]	2.12
**'Fusobacterium sp. MF0224.fa'**	*Fusobacterium perfoetens* ATCC 29250	15.7	[12.8 - 19.2]	20.2	[18.0 - 22.6]	15.7	[13.2 - 18.6]	2.21
**'Fusobacterium sp. MF0224.fa'**	*Piscibacter tructae* 9N8	13.3	[10.5 - 16.6]	19.8	[17.6 - 22.2]	13.6	[11.2 - 16.4]	1.2
**'Fusobacterium sp. MF0224.fa'**	*Fusobacterium pseudoperiodonticum* KCOM 1259T	13.3	[10.5 - 16.6]	19.6	[17.4 - 22.0]	13.6	[11.2 - 16.4]	0.13
**'Fusobacterium sp. MF0224.fa'**	*Fusobacterium nucleatum* subsp. *fusiforme* ATCC 51190	13.2	[10.5 - 16.5]	19.2	[17.1 - 21.6]	13.5	[11.2 - 16.3]	0.99
**'Fusobacterium sp. MF0224.fa'**	*Fusobacterium gastrosuis* CDW1	13.5	[10.7 - 16.8]	19.1	[16.9 - 21.5]	13.8	[11.4 - 16.6]	0.01
**'Fusobacterium sp. MF0224.fa'**	*Fusobacterium hwasookii* KCTC 5108	13.2	[10.4 - 16.5]	19.0	[16.8 - 21.4]	13.5	[11.1 - 16.3]	1.25
**'Fusobacterium sp. MF0224.fa'**	*Hypnocyclicus thermotrophus* DSM 100055	12.8	[10.1 - 16.1]	18.9	[16.7 - 21.3]	13.2	[10.8 - 15.9]	3.4
**'Fusobacterium sp. MF0224.fa'**	*Fusobacterium necrogenes* NCTC 10723	13.6	[10.8 - 16.9]	18.8	[16.7 - 21.2]	13.9	[11.5 - 16.7]	0.98
**'Fusobacterium sp. MF0224.fa'**	*Fusobacterium varium* NCTC 10560	13.5	[10.8 - 16.8]	18.7	[16.5 - 21.0]	13.8	[11.4 - 16.6]	1.14
**'Fusobacterium sp. MF0224.fa'**	*Fusobacterium simiae* DSM 19848	13.0	[10.3 - 16.3]	18.3	[16.2 - 20.7]	13.4	[11.0 - 16.1]	1.13
**'Fusobacterium sp. MF0224.fa'**	*Fusobacterium nucleatum* subsp. *polymorphum* ATCC 10953	13.3	[10.5 - 16.6]	18.0	[15.9 - 20.4]	13.6	[11.2 - 16.3]	1.37
**'Fusobacterium sp. MF0224.fa'**	*Cetobacterium ceti* ATCC 700028	13.4	[10.7 - 16.8]	18.0	[15.9 - 20.4]	13.7	[11.3 - 16.5]	0.05
**'Fusobacterium sp. MF0224.fa'**	*Fusobacterium russii* ATCC 25533	13.0	[10.3 - 16.3]	17.8	[15.7 - 20.1]	13.3	[11.0 - 16.1]	0.45
**'Fusobacterium sp. MF0224.fa'**	*Clostridium rectum* DSM 1295	13.4	[10.6 - 16.7]	17.7	[15.6 - 20.0]	13.6	[11.3 - 16.4]	0.83
**'Fusobacterium sp. MF0224.fa'**	*Anaeromonas gelatinilytica* D2Q-14T	12.5	[9.8 - 15.8]	14.8	[12.8 - 17.0]	12.9	[10.6 - 15.6]	0.61

Table 4: Strains in your dataset

Joint dataset of automatically determined closest type strains (if this mode was chosen), manually selected type strains (if selected accordingly) and the provided user strains, if provided (marked in yellow).

Strain	Authority	Other deposits	Synonyms	Base pairs	Percent G+C	No. proteins	Goldstamp	Bioproject accession	Biosample accession	Assembly accession	IMG OID
Fusobacterium simiae DSM 19848	Slots and Potts 1982	7511 R2-13; ATCC 33568; CCUG 16798	*Fusobacterium simiae*	2465 772	27.1	2263		PRJNA884868	SAMN31056805	GCA_026089295	
Fusobacterium necrogenes NCTC 10723	(Weinberg et al. 1937) Moore and Holdeman 1970	ATCC 25556; CCUG 4949	*Bacillus necrogenes*; *Fusobacterium necrogenes*	2015 236	29.2	1801	Gp0384309	PRJEB6403	SAMEA3649021	GCA_900450765	
Fusobacterium varium NCTC 10560	(Eggerth and Gagnon 1933) Moore and Holdeman 1969	ATCC 8501; CCUG 4858; DSM 19868; JCM 6320	*Bacteroides varius*; *Fusobacterium varium*	3302 398	29.3	3398	Gp0445033	PRJEB6403	SAMEA24554668	GCA_900637705	
Fusobacterium russii ATCC 25533	(Hauduroy et al. 1937) Moore and Holdeman 1970		*Bacteroides russii*; *Fusobacterium russii*	1935 478	28.6	1761	Gp0018673	PRJNA185225	SAMN02441328	GCA_000381725	2519899634
Anaeromonas gelatinilytica D2Q-14T	Zhang et al. 2022	KCTC 15986; MCCC 1K04634	*Anaeromonas gelatinilytica*	2884 908	28.8	2756		PRJNA224116	SAMN13495959	GCF_016820635	
Clostridium rectum DSM 1295	(Heller 1922) Holdeman and Moore 1972	NCIMB 10651; ATCC 25751; JCM 1412; NCIB 10651	*Clostridium rectum*; *Hiblerillus rectus*	2641 807	29.0	2644	Gp0538753				2928356456
Fusobacterium hominis NSJ-57	Liu et al. 2022	CGMCC 1.32833; KCTC 25145	*Fusobacterium hominis*	2072 767	29.1	1926		PRJNA224116	SAMN15815158	GCF_014337255	
Leptotrichia trevisanii DSM 22070	Tee et al. 2002	ATCC 700907; "Wee Tee" 1999	*Leptotrichia trevisanii*	2845 741	30.3	2703	Gp0013905	PRJNA195791	SAMN02441112	GCA_000482505	2528311094

Strain	Authority	Other deposits	Synonyms	Base pairs	Percent G+C	No. proteins	Goldstamp	Bioproject accession	Biosample accession	Assembly accession	IMG OID
Alkalihalobacterium bogoriense ATCC BAA-922	(Vargas et al. 2005) Joshi et al. 2022	LBB3; LMG 22234; MG 22234	*Alkalihalobacillus bogoriensis*; *Alkalihalobacterium bogoriense*; *Bacillus bogoriensis*	4999600	36.7	4822	Gp0044074	PRJNA223013	SAMN02743995	GCA_000621445	2556921655
Fusobacterium perfoetens ATCC 29250	(Tissier 1905) Moore and Holdeman 1973		*Coccobacillus perfoetens*; *Fusobacterium perfoetens*	2103348	26.0	1942	Gp0044079	PRJNA223056	SAMN02743999	GCA_000622245	2556921626
Hypnocyclicus thermotrophus DSM 100055	Roalkvam et al. 2015	JCM 30901; IR-2	*Hypnocyclicus thermotrophus*	2213834	24.8	2045	Gp0325704	PRJNA519327	SAMN10864767	GCA_004365575	2802429728
Cetobacterium ceti ATCC 700028	Foster et al. 1996	NCIMB 703026; M-3333; NCFB 3026	*Cetobacterium ceti*	2576700	28.2	2511	Gp0088813	PRJNA245572	SAMN02745174	GCA_900167275	2585428148
Fusobacterium nucleatum subsp. *polymorphum* ATCC 10953	(ex Knorr 1922) Dzink et al. 1990	CCUG 9126; DSM 20482; JCM 12990; NCTC 10562	*Fusobacterium nucleatum* subsp. *polymorphum*; *Fusobacterium polymorphum*	2440231	26.8	2373	Gp0001475	PRJNA17261	SAMN02469324	GCA_000153625	641736256
Fusobacterium nucleatum subsp. *fusiforme* ATCC 51190	(ex Veillon and Zuber 1898) Gharbia and Shah 1992	CIP 104878; CCUG 32880; DSM 19508; JCM 11024; NCTC 11326	*Bacillus fusiformis*; *Fusobacterium fusiforme*; *Fusobacterium nucleatum* subsp. *fusiforme*	1837112	27.2	1821	Gp0022782	PRJNA167963	SAMN02469584	GCA_000279975	2519899747
Fusobacterium gastrosuis CDW1	De Witte et al. 2017	DSM 101753; LMG 29236	*Fusobacterium gastrosuis*	1816688	28.2	1747		PRJEB15051	SAMEA4368857	GCA_900095285	
Piscibacter tructae 9N8	Takeuchi and Sugahara 2025	DSM 118263; NBRC 116745	*Piscibacter tructae*	2614747	29.4	2364		PRJDB18020	SAMD00794669	GCA_055383505	
Fusobacterium pseudoperiodonticum KCOM 1259T	Park et al. 2019	213; KCTC 5677; JCM 33009; ChDC F213	*Fusobacterium pseudoperiodonticum*	2413057	28.1	2211	Gp0358870	PRJNA416419	SAMN07956184	GCA_002761955	

Strain	Authority	Other deposits	Synonyms	Base pairs	Percent G+C	No. proteins	Goldstamp	Bioproject accession	Biosample accession	Assembly accession	IMG OID
Fusobacterium hwasookii KCTC 5108	Cho et al. 2015	JCM 30218; ChDC F128; KCOM 1249	*Fusobacterium hwasookii*	2356 234	26.9	2200	Gp0036847	PRJNA171181	SAMN02469578	GCA_000292935	2565956808
Fusobacterium sp. MF0224.fa				2021 940	28.2	1902					

Methods, Results and References

The genome sequence data were uploaded to the Type (Strain) Genome Server (TYGS), a free bioinformatics platform available under https://tygs.dsmz.de, for a whole genome-based taxonomic analysis [1]. The analysis also made use of recently introduced methodological updates and features [2,3]. Information on nomenclature, synonymy and associated taxonomic literature was provided by TYGS's sister database, the List of Prokaryotic names with Standing in Nomenclature (LPSN, available at https://lpsn.dsmz.de) [2,3]. The results were provided by the TYGS on 2026-03-17. The TYGS analysis was subdivided into the following steps:

Determination of closely related type strains

Determination of closest type strain genomes was done in two complementary ways: First, all user genomes were compared against all type strain genomes available in the TYGS database via the MASH algorithm, a fast approximation of intergenomic relatedness [4], and, the ten type strains with the smallest MASH distances chosen per user genome. Second, an additional set of ten closely related type strains was determined via the 16S rDNA gene sequences. These were extracted from the user genomes using RNAmmer [5] and each sequence was subsequently BLASTed [6] against the 16S rDNA gene sequence of each of the currently 24311 type strains available in the TYGS database. This was used as a proxy to find the best 50 matching type strains (according to the bitscore) for each user genome and to subsequently calculate precise distances using the Genome BLAST Distance Phylogeny approach (GBDP) under the algorithm 'coverage' and distance formula d_5 [7]. These distances were finally used to determine the 10 closest type strain genomes for each of the user genomes.

Pairwise comparison of genome sequences

For the phylogenomic inference, all pairwise comparisons among the set of genomes were conducted using GBDP and accurate intergenomic distances inferred under the algorithm 'trimming' and distance formula d_5 [7]. 100 distance replicates were calculated each. Digital DDH values and confidence intervals were calculated using the recommended settings of the GGDC 4.0 [2,7].

Phylogenetic inference

The resulting intergenomic distances were used to infer a balanced minimum evolution tree with branch support via FASTME 2.1.6.1 including SPR postprocessing [8]. Branch support was inferred from 100 pseudo-bootstrap replicates each. The trees were rooted at the midpoint [9] and visualized with PhyD3 [10].

Type-based species and subspecies clustering

The type-based species clustering using a 70% dDDH radius around each of the 18 type strains was done as previously described [1]. The resulting groups are shown in Table 1 and 4. Subspecies clustering was done using a 79% dDDH threshold as previously introduced [11].

Results

Type-based species and subspecies clustering

The resulting species and subspecies clusters are listed in Table 4, whereas the taxonomic identification of the query strains is found in Table 1. Briefly, the clustering yielded 17 species clusters and the provided query strains were assigned to 1 of these. Moreover, user strains were located in 1 of 17 subspecies clusters.

Figure caption SSU tree

Figure 1. Tree inferred with FastME 2.1.6.1 [8] from GBDP distances calculated from 16S rDNA gene sequences. The branch lengths are scaled in terms of GBDP distance formula d_5. The numbers above branches are GBDP pseudo-bootstrap support values > 60 % from 100 replications, with an average branch support of 88.9 %. The tree was rooted at the midpoint [9].

Figure caption genome tree

Figure 2. Tree inferred with FastME 2.1.6.1 [8] from GBDP distances calculated from genome sequences. The branch lengths are scaled in terms of GBDP distance formula d_5. The numbers above branches are GBDP pseudo-bootstrap support values > 60 % from 100 replications, with an average branch support of 38.4 %. The tree was rooted at the midpoint [9].

References

[1] Meier-Kolthoff JP, Göker M. TYGS is an automated high-throughput platform for state-of-the-art genome-based taxonomy. Nat. Commun. 2019;10: 2182. DOI: 10.1038/s41467-019-10210-3

[2] Meier-Kolthoff JP, Sardà Carbasse J, Peinado-Olarte RL, Göker M. TYGS and LPSN: a database tandem for fast and reliable genome-based classification and nomenclature of prokaryotes. Nucleic Acid Res. 2022;50: D801–D807. DOI: 10.1093/nar/gkab902

[3] Freese HM, Meier-Kolthoff JP, Sardà Carbasse J, Afolayan AO, Göker M. TYGS and LPSN in 2025: a Global Core Biodata Resource for genome-based classification and nomenclature of prokaryotes within DSMZ Digital Diversity. Nucleic Acid Res. 2025, gkaf1110. DOI: 10.1093/nar/gkaf1110

[4] Ondov BD, Treangen TJ, Melsted P, et al. Mash: Fast genome and metagenome distance estimation using MinHash. Genome Biol 2016;17: 1–14. DOI: 10.1186/s13059-016-0997-x

[5] Lagesen K, Hallin P. RNAmmer: consistent and rapid annotation of ribosomal RNA genes. Nucleic Acids Res. Oxford Univ Press; 2007;35: 3100–3108. DOI: 10.1093/nar/gkm160

[6] Camacho C, Coulouris G, Avagyan V, Ma N, Papadopoulos J, Bealer K, et al. BLAST+: architecture and applications. BMC Bioinformatics. 2009;10: 421. DOI: 10.1186/1471-2105-10-421

[7] Meier-Kolthoff JP, Auch AF, Klenk H-P, Göker M. Genome sequence-based species delimitation with confidence intervals and improved distance functions. BMC Bioinformatics. 2013;14: 60. DOI: 10.1186/1471-2105-14-60

[8] Lefort V, Desper R, Gascuel O. FastME 2.0: A comprehensive, accurate, and fast distance-based phylogeny inference program. Mol Biol Evol. 2015;32: 2798–2800. DOI: 10.1093/molbev/msv150

[9] Farris JS. Estimating phylogenetic trees from distance matrices. Am Nat. 1972;106: 645–667.

[10] Kreft L, Botzki A, Coppens F, Vandepoele K, Van Bel M. PhyD3: A phylogenetic tree viewer with extended phyloXML support for functional genomics data visualization. Bioinformatics. 2017;33: 2946–2947. DOI: 10.1093/bioinformatics/btx324

[11] Meier-Kolthoff JP, Hahnke RL, Petersen J, Scheuner C, Michael V, Fiebig A, et al. Complete genome sequence of DSM 30083^T, the type strain (U5/41^T) of *Escherichia coli*, and a proposal for delineating subspecies in microbial taxonomy. Stand Genomic Sci. 2014;9: 2. DOI: 10.1186/1944-3277-9-2
